# Role of Type I and II Interferons in Colorectal Cancer and Melanoma

**DOI:** 10.3389/fimmu.2017.00878

**Published:** 2017-07-26

**Authors:** Simone Di Franco, Alice Turdo, Matilde Todaro, Giorgio Stassi

**Affiliations:** ^1^Cellular and Molecular Pathophysiology Laboratory, Department of Surgical, Oncological and Stomatological Sciences, University of Palermo, Palermo, Italy; ^2^Central Laboratory of Advanced Diagnosis and Biomedical Research (CLADIBIOR), University of Palermo, Palermo, Italy; ^3^DiBiMIS, University of Palermo, Palermo, Italy

**Keywords:** interferon, cancer, colon, melanoma, tumor immunology, cancer progression, anti-cancer therapy

## Abstract

Cancer can be considered an aberrant organ with a hierarchical composition of different cell populations. The tumor microenvironment, including the immune cells and related cytokines, is crucial during all the steps of tumor development. In particular, type I and II interferons (IFNs) are involved in a plethora of mechanisms that regulate immune responses in cancer, thus balancing immune escape *versus* immune surveillance. IFNs are involved in both the direct and indirect regulation of cancer cell proliferation and metastatic potential. The mutational background of genes involved in IFNs signaling could serve as a prognostic biomarker and a powerful tool to screen cancer patients eligible for checkpoint blocking therapies. We herewith describe the latest findings regarding the contribution of IFNs in colorectal cancer and melanoma by researching their dual role as either tumor promoter or suppressor, in diverse tumor types, and microenvironmental context. We are reporting the most innovative and promising approaches of IFN-based therapies that have achieved considerable outcomes in clinical oncology practice and explain the possible mechanisms responsible for their failure.

## Introduction

Cancer cells originate from healthy cells following genetic or epigenetic changes (1). During tumor development, a crucial role is played by the tumor microenvironment, which is mainly characterized by the presence of stromal and immune cells, as well as by the cytokines produced by each cell subset (2). The cross-talk among these cell types is fundamental for both primary tumor formation and the metastatic process, thus affecting all the steps of carcinogenesis. Previous studies have shown that different immune cell populations and molecules (3–7), play a key role in tumor progression. In this context, type I (α and β) and II (γ) interferons (IFNs) are of particular importance in cancer (8). The immune system is able to recognize not only the self *versus* non-self/pathogen, but also self *versus* transformed cells. This principle was initially proposed by Burnet and Thomas in the 1950s, who suggested the role of the immune system in protecting the host against cancer initiation (9, 10), which then led to the definition of “cancer immunosurveillance.” Nowadays, this model has been confirmed by studies in mouse models and also clinical data on humans. The role of the immune system in tumor progression has been investigated by observing different immunogenic tumor phenotypes grown in immunocompetent/immunodeficient hosts. Indeed, the immune system can have both a negative or a positive effect on tumor growth. It can protect the host or promote tumor onset in different phases of tumor progression, in a process called “cancer immunoediting.” For this reason, it is crucial to study and define all the possible pathways involved in the cross-talk between cancer and immune cells. Cancer immunoediting consists of three phases: (i) the elimination, which is based on the recognition of tumor cells and their being killed by an innate or adaptive immune system (cancer immunosurveillance model); (ii) the persistence, based on the failure of the elimination step that leads to an equilibrium between growing cancer cells and immune system pressure; and (iii) the escape, which starts when the cancer cell growth is able to overcome the protective effect of the immune system due to immune exhaustion/inhibition or the generation/selection of resistant cancer cell clones. Schreiber and colleagues published an excellent review about the role of IFNs in the different steps of anti-tumor immunity in 2006 (8).

The interaction between cancer and immune cells is finely controlled during all the stages of tumor development, in which the IFNs definitely assume a pivotal role. Immunotherapy has already achieved impressive results especially in immunogenic tumors, such as melanoma, which is characterized by a high number of DNA mutations responsible for the creation of neo-antigens recognized by immune cells (11). Colorectal cancer (CRC) is generally considered a scarcely immunogenic tumor but, recently, increased attention has been given to CRCs with defects in miss match repair (MMR) mechanisms (12). These tumors account for approximately 15% of the total cases and they are usually associated with a favorable prognosis at early stages while if metastatic or relapsed, they do not do well (13). MMR-deficient CRCs display a high lymphocyte infiltrate that is engaged by the conspicuous number of neo-antigens expressed on the surface of CRC cells and that contributes to cancer clearance. Thus, the treatment of MMR-deficient CRCs with immunotherapy has improved their therapeutic outcome (12) and may represent a crucial clinical challenge for all aggressive CRCs.

Being aware that dealing with all the aspects of IFN biology in cancer may result reductive, this review presents a comprehensive overview of the latest findings regarding IFN cell signaling and its clinical administration as non-specific immunotherapy, with particular attention given to CRC and melanoma.

## IFNs’ Role in Cancer

The IFNs are cytokines that are released in the presence of pathogens or cancer cells. They are involved in many biological processes spanning from cellular immune response against viral/microbial infections to cell cycle, differentiation, and apoptosis (8, 14). IFNs are divided into three subgroups: type I (α, β, ε, κ, and ω), binding IFNα/β receptor 1 (IFNAR1) and IFNAR2 subunits, type II (γ) that binds IFN-γ receptor 1 (IFNGR1), and type III (λ), which binds the IFN-λ receptor 1 and IL10 receptor subunit β heterodimeric receptor (14). Dendritic cells (DCs) are the main IFN-α producing cells however, many other cells such as infiltrating innate immune cells, can produce it in an autocrine or paracrine manner. IFN-β is usually produced in an autocrine manner to limit proliferation stimuli as a negative feedback loop. Other type I IFNs, including ε, κ, and ω, are less characterized and their expression seems to be tissue/disease specific. The type II IFNs are mainly released by γδ T cells and natural killers (NKs). Following the binding of IFNs to their receptors, associated with JAK1 and TYK2, they are phosphorylated, thus leading to the activation of STATs that translocate to the nucleus and activate the expression of several target genes.

As previously mentioned, IFNs can activate a plethora of biological signaling pathways in tumor cells including cell proliferation, differentiation, survival, and invasion. IFNs can indeed affect cell proliferation in tumor cells both by prolonging or blocking the cell cycle (15, 16), regulating p21 (16), p38 MAPK (17), or CRKL, which in turn interacts with RAP1A, a tumor suppressor that antagonizes RAS (18, 19). IFNs can also regulate the apoptotic machinery by controlling the extrinsic and intrinsic apoptotic pathways (20, 21). Thanks to the deletion of type I IFN genes (22) and the down-regulation of IFN receptors (23, 24) or signaling molecules involved in the IFN cascade, such as STAT1 (25), all these regulatory effects can be bypassed by tumor cells. All these findings can explain the partial failure of IFN treatment used to control cancer cell proliferation in different models.

Beyond all the above-mentioned direct effects, IFNs can also indirectly regulate tumor cell growth, affecting different biological processes involved in tumor progression, such as angiogenesis and immunity (26, 27). The first demonstration of an indirect effect has been highlighted by Brouty-Boye and colleagues who showed that the administration of IFNs increased the survival of mice affected by lymphocytic leukemia, regardless of the intrinsic sensibility of tumor cells to IFN preparations (28). Indeed, IFNs behave as activators of several immune cells including macrophages, DCs, NKs, B cells, and T cells. It has recently been demonstrated that DCs producing type I IFNs induce an anti-tumor effect in mice affected by melanoma (29). Contrarily, the accumulation of infiltrating DCs was associated with a poor prognosis in breast cancer (30). These apparently conflicting results can be explained by recent findings, which illustrate that IFN-α-deficient tumor-associated DCs accumulate in aggressive tumors and lead to the expansion of regulatory T cells (T_reg_), which contribute to tumor immune tolerance and a poor clinical outcome (31). It has been demonstrated that tumor cells often abrogate IFN production to successfully metastasize (32). The immunoregulatory effect of IFNs includes the up-regulation of tumor antigens expression (33), the DCs tumor antigen presentation to T cells, the acquisition of CD8+ T cell effector phenotype (34, 35), the down-regulation of (T_reg_) (36, 37), the inhibition of myeloid-derived suppressor cells (MDSCs) accumulation (38) (T_reg_ and MDSCs accumulate in circulation of cancer patients where they negatively regulate the cytotoxic activity of T cells), and the monocyte differentiation in M1-polarized immunostimulatory macrophages (39). Finally, IFNs can increase the major histocompatibility complex (MHC) antigen presentation (40), the expression of ligands involved in immune checkpoints (such as the programmed cell death protein 1, PD-1) (41), and the release of cytokines (42, 43).

## Induction of IFNs in Cancer Immunotherapy

It is well known that cancer cells can act with a multitude of immune evasion processes, whose mechanisms are crucial to make many anti-tumor therapies ineffective (44). However, recent findings suggest that the stimulation of the immune system in cancer patients is sufficient to counteract tumor progression, directly inducing tumor cell death, and indirectly boosting the immune system against it (45). Among the immune targets that show promising results in preclinical and clinical studies, we find the toll-like receptors (TLRs), oncolytic viruses (OVs), and stimulator of interferons genes (STING). Here we describe the anti-tumor properties of the above-mentioned pathways that affect the production of IFNs in cancer cells that in turn cause cell death, as well as their possible application in cancer immunotherapy, alone or in combination with standard anti-tumor regimens.

The first strategy involves the use of TLR agonists. TLRs are mammalian homologs of the toll protein of Drosophila and include 10 members in human (46). Some of these proteins are present on the cell membrane (TLR1, TLR2, TLR4, TLR5, and TLR6), while TLR3, TLR7, TLR8, and TLR9 are expressed on the endosome’s membrane. These receptors share the protein structure, which includes the transmembrane domain, the ectodomain responsible for ligand binding, and the cytosolic toll/IL-1 receptor (TIR) domain (47). The binding of pathogen-associated molecular pattern (PAMP) or damage-associated molecular pattern (DAMP) to the ectodomain triggers the association of TIR with adapter proteins thus leading to the activation of nuclear factor-kB (NF-kB) inflammation pathway, and the release of type I IFNs (47). Different TLRs recognize specific PAMPs and DAMPs, including lipoproteins, peptidoglycans, viral single or double strand RNA, lipopolysaccharides bacterial flagellin, CpG-containing oligodeoxynucleotides, and heat-shock proteins. Several TLR agonists are currently under investigation in preclinical and clinical trials for their use in cancer therapy, as recently reviewed by Shi and colleagues (48). In the tumor context, recent evidences show that TLRs are expressed not only on immune cells, but also on cancer cells (49). TLRs on immune cells act as immune system sensor molecules that detect tumor antigens and start the elimination of cancer cells through the activation of effector cells (50). Thanks to this process, the immune cells avoid the establishment of an inflammatory tumor microenvironment. Contrarily, TLRs expressed on cancer cells enhance immune suppression and favor the establishment of an inflammatory microenvironment, thus leading to tumor evasion from immune surveillance (51, 52). Although the exact mechanisms of action is still unclear, TLR expression on cancer cells indeed correlates with tumor progression, with an increased cancer cell proliferation and invasion index (53).

The use of TLR agonists in combination with standard anti-tumor treatments, including chemo- and radio-therapy has shown promising results. In fact, the combinatorial treatment showed more pronounced cancer cell proliferation inhibitory effect and less side effects than single agents (54, 55). This synergy is probably due to the enhanced DC maturation following treatment with TLR agonists. Chemo- and radio-therapy are in fact sufficient to induce the release of tumor antigens, which in the presence of mature DCs, leading to the antigen presentation, the release of type I IFNs, and the priming of cytotoxic T lymphocytes (CTL).

Oncolytic viruses have recently drawn the attention of the scientific community for their promising application as anti-tumor agents in cancer immunotherapy. The OVs are defined as wild-type, or genetically engineered viruses, which are able to selectively replicate into cancer cells thus inducing cell death, without affecting normal cells. The rational for the use of OVs in cancer immunotherapy lies on previous observations about tumor regression following systemic viral infection (56). Several clinical trials have been performed between 1950 and 1980 in order to understand if and how viral infection could be used for cancer treatment. The main problem in those studies at that time was represented by the inability to limit the viral replication in cancer cells. Thanks to today’s knowledge regarding virus replication and the innovative strategies to manipulate the virus genome, in the last two decades it was possible to use OVs in clinical settings with very important results in clinical trial. OVs belong to two important classes: the viruses that preferentially replicate in cancer cells due to their sensitivity to innate antiviral agents and their dependence on oncogenic signaling pathways (parvoviruses, myxoma virus, reovirus), and genetically modified viruses to be used as vaccine vectors (measles virus, poliovirus, vaccinia virus) or genetically engineered viruses that bear mutations, which make them optimal for replication in cancer but not in healthy cells (adenovirus, herpes simplex virus, vescicular stomatitis virus) (57, 58). There are several advantages to using OVs compared with standard anti-tumor regimens, among these we find (i) the absence of acquired resistance (which is one of the most common issues when using standard treatments); (ii) the tumor selectivity and the low grade side effects; (iii) the virus’ replication that increase virus copies over time (contrary to the normal pharmacokinetics of conventional drugs that decrease over time); and (iv) the possibility to deliver viruses and control their effects thus leading to high therapeutic indexes (i.e., blocking virus neutralization, increasing stability, and delivery with nanoparticles). The main goal in the use of OVs for cancer immunotherapy is the induction of direct or indirect (by activating immune cells) death of cancer cells. The most important issue that researchers are facing by using OVs for cancer immunotherapy is due to the induction of inflammatory process in the tumor context. Inflammation can play a dual role in tumor progression, leading to anti-tumor immunity on one side and, if chronic, promoting tumorigenesis, and inhibiting T cell anti-tumor activity. For this reason, a better understanding of the immune response following OV treatment is crucial for the development of the next OVs-based immunotherapeutic strategies. Another important issue to be addressed is represented by the expression of immune evasion genes in OVs. To solve this problem, several mutations have been studied to improve the induction of immunity and the presentation of tumor-associated antigens. However, this could lead to a decreased virus replication and spread. Interestingly, following OV-mediated cell death, cancer cells release tumor-associated antigens, viral PAMPs, DAMPs, and cytokines (including type I IFNs), thus leading to the maturation of antigen-presenting cells (APCs) such as DCs.

Several clinical trials have been performed with OVs as a cancer immunotherapy agent, mostly ended in phase I and phase II. The most promising is represented by the HSV1 expressing GM-CSF, which showed very important results in phase III, in the treatment of unresected stage IIIB-IV melanoma (T-Vec). This OV has been engineered to have a double mutation in the γ34.5 and α47 genes (for cancer-selective replication and enhanced anti-tumor response, respectively), and the insertion of the GM-CSF human gene to enhance the anti-tumor immunity induction. This trial includes subjects treated with intratumor injections of T-Vec of GM-CSF alone, reporting 16% of response rate for T-Vec arm, compared with 2% of response rate for the patients treated with GM-CSF alone (59). T-Vec was recently approved by the FDA in United States, Europe, and Australia for the treatment of melanoma patients.

Another strategy to increase IFN production in the tumor context is to act on the STING pathway (60–62). STING is a transmembrane protein of the endoplasmic reticulum activated by the presence of double strand DNA in the cytosol and acts as defense mechanism against viral, bacterial, or mitochondrial DNA that can be detected by the host immune system. Cytosolic DNA is detected following its binding with cyclic-GMP-AMP synthase, which produces cyclic GMP-AMP (cGAMP) from guanosine triphosphate and adenosine triphosphate. cGAMP plays a crucial role in the binding and activation of STING (63, 64). The activation of STING leads to a cascade that ends with the phosphorilation of interferon regulatory factor 3 (IRF3), whose translocation into the nucleus is crucial in driving the transcription of IFN-β, as well as other target genes (65). The importance of the STING pathway in the production of IFNs, activators of immune system cells (i.e., CD8+ T cells), has not only been demonstrated in infectious diseases, but also in cancer (62). The STING pathway is able to drive cancer cell death also in an IFN-independent manner, through the association with Bcl-2-associated X protein on mitochondria thus inducing the mitoptosis caspase 9- and 3-dependent (66–68). It has been demonstrated that apoptotic caspases are also able to suppress the STING pathway as negative feedback (69). A better understanding of the regulation mechanisms could lead to the design of an optimal strategy for STING’s use in clinical setting in the near future.

## IFNs and CRC

CRC is the third most common cancer and the fourth cause of cancer-related death, with more than one million new diagnoses made every year (World Cancer Report February 2015). CRC progression is characterized by the transformation of normal mucosa into an adenoma and then into a malignant tumor. It is a very slow process that involves the acquisition of multiple mutations that give tumor cells an advantage in cell proliferation and migration. Recent findings show that tumor cells originate from healthy stem cells, thus generating the so called cancer stem cells (CSCs) (1, 70, 71). This hierarchical carcinogenesis model is important because it provides an explanation for tumor heterogeneity. CSCs are responsible for chemoresistance and relapse, being characterized by self-renewal capabilities, multi-lineage differentiation capacity, enhanced DNA repair machinery, and high expression levels of anti-apoptotic proteins and ATP-binding cassette (ABC) transporters (72). Despite the fact that all these properties seem to be intrinsically owned by CSCs, the tumor microenvironment, including immune cells and the cytokines they produce, can play a crucial role in maintaining “cancer stemness,” as well as regulating differentiation and apoptotic index (73).

**Figure 1:**
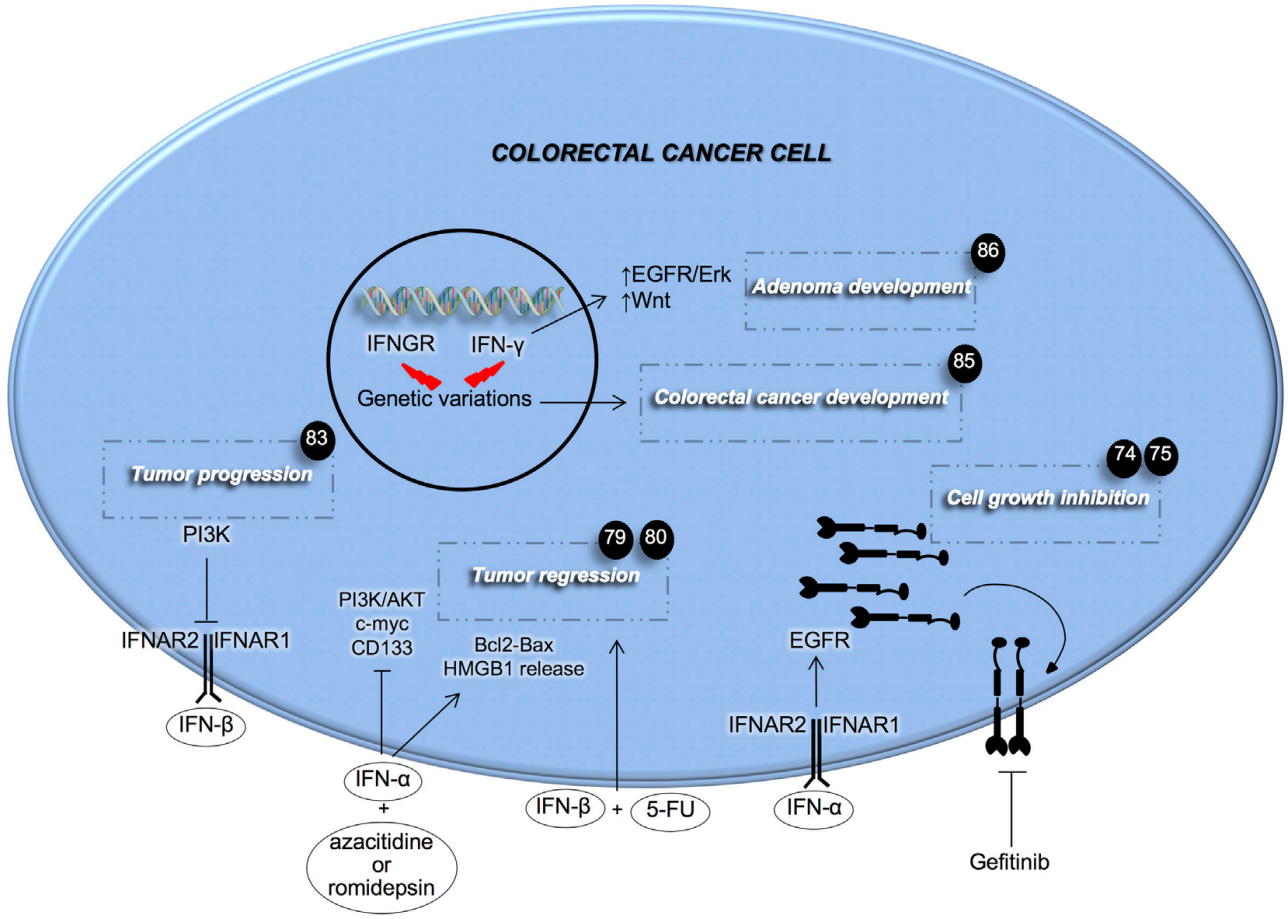
Effects of type I and II interferons (IFNs) on colorectal cancer cells. IFNs of type I aid anti-cancer therapy in inhibiting cancer cell growth. PI3K activity inhibits the signaling of IFN-β. Genetic mutations in both IFN-γ and its receptor induce adenoma and colorectal cancer development. Fluorouracil (5-FU), high mobility group box 1 (HMGB1), interferon-α/β receptor (IFNAR).

Recent findings have highlighted the importance of the IFN signaling pathway in CRC (Figure 1). IFNs’ mechanisms of action are numerous. Goldstein and colleagues investigated the role that IFN-α has in regulating the EGF pathway in CRC (74). Here, the authors demonstrated that treatment with IFN-α increases the expression of EGFR on both the cell’s surface and endocytic vesicles. The latter phenomenon was accompanied by a marked growth inhibition (74). This result paved the way for a combinatorial treatment with repeated IFN-α administration followed by EGFR inhibition to completely eradicate CRC. Preclinical data showed that the combination of IFN-α and the EGFR tyrosine kinase inhibitor, gefitinib, slowed down the growth of head and neck xenografts in nude mice, and prolonged mice survival (75). A clinical trial conducted on metastatic renal carcinoma demonstrated the efficacy of the kinase inhibitor sorafenib with IFN-α in ameliorating the overall response and disease stabilization (76). mTOR is a downstream effector of the EGFR pathway and its targeting with temsirolimus, the first-line therapy for renal cancer, coupled with IFN-α, did not succeed in improving overall survival (OS) of patients (77). Several explanations have been postulated such as the occurrence of side effects and a decreased temsirolimus dose when compared with single treatment. However, the achievements obtained with EGFR signaling inhibition and concomitant IFN-α administration, seem to promise improvement to current therapies and warrant further investigation.

IFN-α was also studied for its involvement in the regulation of angiogenesis in CRC. Fidler’s group showed that systemic administration of IFN-α can inhibit liver metastases and cause a strong reduction in tumor growth, vascularization, and bFGF and MMP9 expression (78). This effect seems to be due to the induction of apoptosis in metastases-associated liver endothelial cells. Moreover, recently it was reported that IFN-α treatment in combination with methyltransferase and histone deacetylase inhibitors, could have a very promising therapeutic potential, inducing both an antiproliferative and pro-apoptotic effect on metastatic colorectal CSCs (CR-CSCs) (79). This combinatorial regimen is also able to induce the release of high mobility group protein B1 by CR-CSCs, thus inducing the so called immunogenic cell death (79).

The other type I IFN, the IFN-β, was studied for its role in the CRC model. It has recently been shown that IFN-β can sensitize CRC cells to 5-FU treatment with a potent effect on the reduction of tumor mass, suggesting a novel strategy to selectively target CRC (80). In line with recent findings, which show that the PI3K pathway is crucial for CSCs ability to proliferate and invade distant organs (81, 82), Spitz and colleagues showed that activating this pathway is an important contributor to IFN-β treatment resistance (83).

Slattery and colleagues showed that genetic variations in *IFN-*γ, specifically in IFNGR or IRFs, are associated with the increased risk of developing CRC and decreased survival after diagnosis (84, 85). In particular, the authors demonstrated that the *IRF2* mutational status is associated with both colon and rectal cancer, whereas mutations in other genes involved in IFN signaling pathway were uniquely associated with colon (*IFN-*γ and *IRF3*) or rectal cancer (*IFNGR1, IFNGR2, IRF4, IRF6*, and *IRF8*). Accordingly, Qu and colleagues showed that the deficiency of endogenous IFN-γ in adenomatous polyposis coli-mediated intestinal tumor, increased the number and size of adenomas. Moreover, the authors found that these effects were driven by increased EGFR/Erk and Wnt pathways. The administration of IFN-γ led to the inhibition of CRC cell proliferation, while the knockdown of IFNGR1 stimulated cell proliferation and colony formation potential (86). Interestingly, the use of IFN-γ in the treatment of CRC has recently shown important results, against the CSC subset by inducing apoptosis both in *in vitro* and *in vivo* (87). Huang and colleagues also demonstrated that treatment with IFN-γ has a synergistic effect when combined with the conventional oxaliplatin treatment in eliminating both CSCs and differentiated CRC cells (87).

The evidence, which shows that not all the cancer patients respond uniformly to the treatment with IFNs, encourages the researchers to find possible predictive response markers to develop targeted rather than randomized trials in the imminent future.

## The Use of IFNs in the Treatment of CRC

The use of IFN-based treatment has been tested over the last decades on many types of cancer including renal cell carcinoma, breast cancer, melanoma, and CRC. At first glance, the obtained results were not encouraging as they demonstrated a significant regression in only a small number of treated patients. The treatment costs are high and have unpleasant side effects due to enhanced toxicity of combinatorial regimens given by IFNs, thus discouraging researchers. However, the first studies were conducted using only a limited number of patients and without the correct optimization of the regimens. Novel discoveries, which clarified how the effects of IFNs on solid tumors are more likely to be dependent on immune cells rather than having a direct effect on tumor cells, have permitted to obtain very promising results in the treatment of cancer patients using IFNs. Reason for which these molecules have been approved for the treatment of tumors. In fact, several innovative formulations of IFNs have recently been used in the clinic (88). For instance, the two most important IFN-based treatments consist in the use of pegylated IFNs or agonists of STING pathway. IFNs, such as other small protein drugs, have a relatively short half-life, thus requiring continuous treatment and often having limited efficacy. Pegylation, which is the addition of poly ethylene glycol, increases IFNs’ stability and reduces their toxicity, leading to the increment of both pharmacokinetics and efficacy of IFNs in treating several diseases, including CRC. A recent study has indeed shown that pegylated IFN-β possesses anti-tumor activity in colon xenografts models (89). Baker and colleagues demonstrated that the combinatorial use of pegylated IFN-β and bevacizumab has a greater tumor growth inhibition effect compared with exclusively using pegylated IFN-β, which had no significant effects compared with vehicle control (89).

The protective role of STING against CRC has been recently demonstrated (90–92). Barber and colleagues have shown that STING is fundamental for the induction of inflammatory wound repair and the deregulation of IL-22BP by IL-18 (90). Moreover, they have found that loss of STING could enable cancer cells to evade the host immunosurveillance processes, due to the absence of key cytokines that facilitate anti-tumor-T cell priming (91). Kanneganti and colleagues demonstrated that the absence of STING was sufficient to increase the production of the pro-inflammatory cytokines IL-6 and keratinocyte chemoattractant, due to the abrogation of NF-kB and STAT3 signaling pathways (92). However, the role of STING in tumor development is cause for much debate. In another recent study, Barber’s group has shown that the absence of STING makes mice resistant to DMBA-induced skin cancer (93). This finding can be explained by the absence of pro-inflammatory cytokines, which are crucial players in the inflammation-induced carcinogenesis in different cancer models.

For all the above-mentioned reasons, STING agonists have attracted the interest of the scientific community and they have been used on humans in combination with standard chemotherapy.

Unfortunately, before the discovery that the analog of flavone acetic acid (DMXAA) was a potent agonist of STING, a phase 3 clinical trial enrolling advanced non-small cell lung cancer patients was not effective in improving neither OS nor progression-free survival (94). The reason for this failure was explained a few years later with the finding that DMXAA was specific for mouse STING and not for the human protein (95–97). So Gajewski and colleagues decided to synthesize a large panel of cyclic dinuocleotide (CDN) derivatives able to activate both mouse and human STING, without significant toxicity. They showed that intratumoral injection of selected CDNs into established xenograft derived by subcutaneous injection of CT26 cells into mice left flanks was able to greatly reduce tumor growth and promote lasting systemic antigen-specific T cell immunity (60).

Recently, a phase I clinical trial was opened using a human agonist of the STING pathway for patients affected by solid tumors (NCT02675439).

## Innate and Adaptive Immunity Cooperates in Either the Eradication or Promotion of Melanoma through Type I and Type II IFNs

Melanoma accounts for more than 1/100.000 new case per year worldwide and its incidence is increasing especially among light-skinned ethnicities (98). It represents the most aggressive skin cancer and is characterized by its life-threatening spread and rapid disease progression. Melanoma is a highly curable cancer if diagnosed in its early stages, while if metastatic, it is unresponsive to conventional anti-cancer therapy and has less than a 20% of 5-year survival rate (99). Melanoma originates from a malignant transformation of melanocytes. These are cells that during embryonic development migrate from the neural crest and move to the skin where they differentiate and start producing pigment (100). Therefore, melanoma cells possess intrinsic capabilities to migrate and to be plastic, switching their phenotype in accordance to the hostile cancer milieu (101). Several recent findings reported that such a plastic behavior is guided by a small sub population of stem-like cells that were prospectively isolated for the expression of CD133, ABCG2 (102), nestin (103), ABCB5 (104), and CD271 (105). On the other hand, Morrison and colleagues noticed that a single melanoma cell transplant in NOD/SCID IL2rg−/− mice, generated xenografts regardless of membrane marker expression (106).

**Figure 2:**
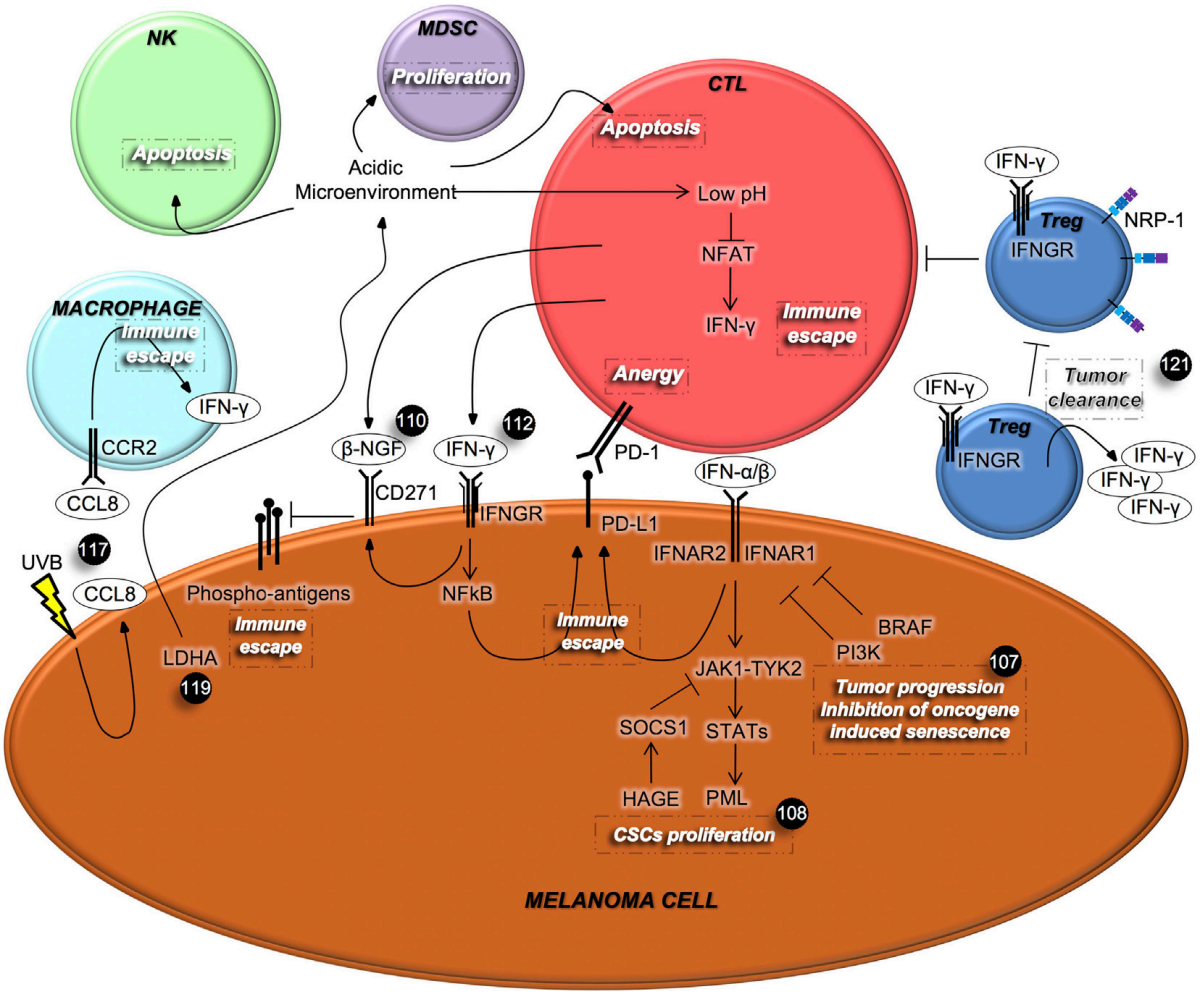
Interferons (IFNs) mediate the cross-talk among melanoma cells and immune cells. IFNs, which are secreted by immune cells, regulate different aspects of cancer cells’ behavior, including proliferation and metastatic spread. In turn, cancer cells can affect immune cell viability and their IFNs’ secretion. β-NGF, β-nerve growth factor; CCL8, chemokine (C-C motif) ligand 8; CCR2, C-C chemokine receptor type 2; CTL, cytotoxic T lymphocyte; IFNGR, interferon-γ receptor; LDHA, lactate dehydrogenase A; MDSC, myeloid-derived suppressor cell; NRP-1, neuropilin-1; NFAT, nuclear factor activated T cells; NK, natural killer; PD-1, programmed cell death protein 1; PD-L1, programmed death-ligand 1; PML, promyelocytic leukemia protein; T_reg_, regulatory T cells; UVB, ultraviolet B.

In order to circumvent melanoma CSC therapy resistance, different therapeutic approaches have been tested, especially those potentiating the immune response against this high immunogenic type of cancer. Some examples are represented by the ectopic administration of type I IFNs, currently being tested in patients affected by melanoma. However, little attention has been given to the endogenous IFN pathway (Figure 2). In this context, it has been demonstrated that *in vitro* and *in vivo* inactivation of IFN signaling by using shIFNAR1 cells and *IFNAR1*-null mice, respectively, overcomes oncogenes-induced senescence, a tumor suppressive signal that protects DNA damaged cells from the onset of cancer. Cancer cells initially proliferate and then become senescent. However, additional events such as mutation in PTEN, PI3K, and mTOR can cause them to abandon their state of senescence. Type I IFNs are produced following DNA damage and contribute to senescence in these cells. IFNAR1 can be partially down-regulated by BRAF activation and additional mutations such as in PI3K, can disrupt this balance and abolish the tumor suppressive role of IFN signaling. Preservation of IFN signaling can protect melanocytes from becoming malignant and renders melanoma cells sensitive to BRAF inhibitors and immunotherapy (107).

As mentioned above, IFN-α binding to its receptors IFNAR1 and IFNAR2, triggers the phosphorylation of Tyr2 and Jak1, which in turn activates the JAK/STAT signaling cascade. In ABCB5+ melanoma cells, STAT is responsible for the transcription of the tumor suppressor promyelocytic leukemia protein, which inhibits proliferation of malignant melanoma initiating cells. However, melanoma cells can overcome the IFN-α effect via suppressor of cytokine signaling 1 (SOCS1), which mediates ubiquitinization and degradation of JAK. Interestingly, the helicase HAGE is selectively expressed on tumor cells and promotes the expression of SOCS1 (108).

Another example of type I IFN involvement in boosting anti-cancer immunity is represented by the recent discovery reporting that *in vivo* growth of melanoma cells is strictly dependent on the production of prostaglandin E2 (PGE_2_) as a result of the cyclooxygenase (COX) activity. PGE_2_ limits the activity of type I IFNs-secreting immune cells with consequent failure of tumor eradication. Based on their preclinical data, the existence of a positive correlation between type 1 IFN signature in melanoma patients and longer relapse-free survival (RFS), authors proposed to couple COX inhibitors to anti-PD-1 therapy in clinical settings (109).

Differently, over-expression of CD271 in melanoma cells is induced by the IFN-γ released from CTLs at tumor sites. CTLs also secrete the CD271 ligand, named β-nerve growth factor (β-NGF), whose binding to its receptor, causes the down-regulation of the antigen expression on the melanoma cells’ surface that leads to the suppression of CTL activation (110). The programmed death-ligand 1 (PD-L1) is physiologically expressed on T, B, and APC. This latter is necessary for normal tissue homeostasis so to guarantee tolerance and protection. PD-L1 is also expressed on non-immune cells such as melanoma cells, and its engagement to its cognate receptor PD-1 on T cells, inhibits T cell proliferation, survival, and cytokines release (111).

Interestingly, PD-L1 is expressed on melanoma cells following stimulation with IFN-γ, through a mechanism known as “adaptive immune resistance,” causing a double suppressive stimuli for CTLs (110). These results were confirmed by Hersey and colleagues who defined the mechanism that modulates the inducible PD-L1 expression. By using both the NF-kB pharmacological inhibitors, BMS-345541 and I-BET151, and siRNA for NF-kB subunits, they have proven that IFN-γ released by tumor-infiltrating lymphocytes up-regulates PD-L1 expression on melanoma cells (112). Moreover, the blockade of PD-1 in a murine model, increased the secretion of IFN-γ and CXCL10 and was critical in recruiting anti-tumoral T cells into tumor sites (113).

These findings led to the hypothesis that melanoma cells activate a self-protective response system against the immune attack in the tumor microenvironment and that patients could benefit from the double combination treatment using targeted therapy (or chemotherapy) and anti-PD-1 immunotherapy. Interestingly, even though melanoma patients, who experienced a resistance to BRAF inhibitors, showed an up-regulation of PD-L1 (114), a large number of studies reported that the inducible PD-L1 expression is not correlated with *BRAF* mutational status (112, 115). On the other hand, the “innate immune resistance” model claims that constitutive PD-L1 expression can be triggered by driver mutations in oncogenes. However, it seems to not be applicable in the case of melanoma, where constitutive PD-L1 expression is not associated with mutations in BRAF, PTEN, NRAS, and AKT amplification (116).

Additionally, the exposure of mouse neonatal skin to ultraviolet B (UVB) radiation caused enhanced survival and immunoevasion of melanoma cells. Upon UVB exposure, melanoma cells start producing chemokine receptor type 2 (CCR2) ligands that recruit CCR2-expressing macrophages to the skin. Macrophages in turn secrete IFN-γ, thus activating melanoma cells to produce chemokine (C-C motif) ligand 8, a CCR2 ligand. This feedback mechanism augments the interaction between melanoma cells and macrophages promoting an inflammatory and pro-tumorigenic microenvironment (117).

Despite the previously mentioned tumor promoting effects of IFN-γ, several studies showed its role as anti-tumor mediator. This pro-inflammatory cytokine is secreted by NK, NKT, and activated T cells and can potentially exerts an anti-tumor response by activating CTLs, monocytes, NK cells, and macrophages, promoting the expression of MHC class I (118). Indeed, some reports divulged that IFN-γ inhibits cancer cell proliferation and angiogenesis as well as enhancing the immune response in melanoma (119). As soon as 3 days after inoculation, IFN-γ-secreting γδ T cells are recruited to the B16 mouse melanoma cell injection site in immunocompetent mice, suggesting that γδ T cells are involved at early stages of immunosurveillance against the development of cancer (120). By using immunocompetent C57BL/6 mice, Kreutz’s group demonstrated that tumor cells with low lactate dehydrogenase A (LDHA) activity, which metabolizes pyruvate in lactate during elevated glucose consumption and is responsible for lowering the intracellular and extracellular pH, grew slower than control cells *in vivo* due to immune surveillance (119). Indeed, control tumors characterized by high LDHA activity, had a reduced or undetectable number of CD8+ T cells and NK cells, respectively. CD8+ T cells were also inactive as they lacked CD25 expression. Contrarily, there was no difference in tumor growth in cells harboring different levels of LDHA in Rag2−/− γc−/− mice, which lack T, B, and NK cells, suggesting the important role played by the immune system. Acidification of the tumor microenvironment caused the apoptosis of T and NK cells. Moreover, it lowered the intracellular pH of T cells, compromising the activity of nuclear factor activated T cells, which controls the transcription of IFN-γ. Mice lacking IFN-γ and IFN-γR1 failed to counteract tumor growth in mouse melanoma cells, possessing both IFN-γ and IFN-γR1, regardless of LDHA status. This suggests the importance of IFN-γ signaling occurring in the immune cell compartment. Low IFN-γ levels impair the switch of MDSCs into APCs, given that the acidic microenvironment provides IL-23 necessary for MDSCs survival. Hence, authors proved that melanoma patients with high LDHA levels possess high extracellular lactate levels and are therefore associated with poor prognosis (119).

Another immune cell compartment that plays a fundamental role in cancer is constituted by T_regs_, which facilitate tumor progression by limiting anti-tumor immunity. Vignali’s group described that neuropilin-1 deficient T_regs_, by secreting IFN-γ, destabilize the function of surrounding wild-type T_regs_ preventing them to exert their pro-tumorigenic activity. Furthermore, IFN-γ-mediated fragility of Tregs is mandatory for the efficacy of anti-PD-1 therapy (121).

## IFN-Based Therapies in the Management of Melanoma

Among the IFNs family of glycoproteins, IFN-α has been the most implicated in clinical settings. IFN-α is administered at high, intermediate, and low doses according to the type of molecule and patient morbidity. IFN-α is mainly given in the adjuvant setting to those patients that possess a high risk of reoccurrence after having undergone a melanoma resection. In 1996, the United States Food and Drug administration approved the use of IFN-α2b for this particular category of patients on the basis of the improved RFS and OS observed in the first clinical trial using high doses of IFN-α2b (ECOG 1684) (122). Two other clinical trials, the E1690 and E1694, compared high doses of IFN to low doses and to vaccines with the ganglioside GMK, respectively. Both clinical trials showed improvements in RFS, while only E1694 showed increased OS (123, 124).

As mentioned earlier, IFN-α2b has been conjugated with polyethylene glycol (Peg-IFN-α2b) to reduce its clearance and augment immunogenicity. It was approved by the FDA in 2011 as an adjuvant therapy for high risk patients affected by melanoma stage II and III. Peg-IFN-α2b has been tested in clinical trial (EORTC 18991) in high risk melanoma patients with involvement of lymph nodes and achieved improved RFS but no differences in OS (125).

Kirkwood and colleagues showed that patients treated with IFN-α2b in the neoadjuvant setting had longer OS (126). Other studies are showing results from combination therapy using IFN-α2b and chemotherapy and fail to show complete responses (127).

Interestingly, both IFN-α2b and Peg-IFN-α2b are being tested in combination with the BRAF inhibitor vemurafenib (NCT01943422 and NCT01708941). IFN-α2b efficacy is increased by administering anti-CTLA4 antibody to patients with unresectable melanoma (NC01708941) and the combination treatment utilizing Peg-IFN-α2b and the anti-PD-1 pembrolizumab are currently in clinical trials (127). In order to overcome the serious side effects of systemic administration of high dose IFN-α, a cell-based therapy has been developed, in which cells are engineered to express IFN-α and to convey it to the tumor site. For this purpose, mesenchymal stem cells (MSCs) are ideal candidates because they are easy to isolate, expand *ex vivo* and transduce with viral or non-viral vector encoding IFN-α. They also possess an excellent aptitude for migrating to inflammatory sites, which is typical of tumor microenvironments. In particular, MSCs tropism in tumors is dictated by the expression of adhesion molecules and receptors that recognize factors secreted by cancer cells (128). However, evidence suggests that MSC therapy should be administered in association with other therapies in order to improve its efficacy. Furthermore, IFN-α directly causes tumor cell apoptosis and impairs tumor vasculature, while the contribution of the immune system is still controversial. Thus, adjusting the number of MSCs and the quantity of IFN-α secreted could eventually potentiate the involvement of the immune system.

Interestingly, a phase I clinical trial currently ongoing in patients with advanced melanoma, is evaluating the efficacy of intravenous delivery of nanoparticles holding RNA. These particles are called RNA-lipoplexes, which encode for tumor antigens and selectively target DCs and macrophages at lymphoids organs. These transduced immune cells start secreting IFN-α following TLR7 stimulation, thus promoting their maturation when they express the epitope of interest needed for T cell priming. Additionally, IFN-α transforms activated T cells into effector T cells (129). This strategy provides an ideal example of cooperation between innate and adaptive responses occurring during tumor eradication. Moreover, being that RNA can encode for a broad range of antigens, it represents a powerful approach in the treatment of various types of cancer.

As in CRC, STING is a sensor for cytosolic DNA and mediates the transcription of IFN-β in melanoma. Intratumoral injection of STING agonists potentiates the secretion of IFN-β by DCs that have been exposed to tumor DNA. This leads to enhanced cross-priming between APCs and T cells. Preclinical data showed that mice treated with STING antagonists showed a reduction of melanoma metastases and durable immune memory (60).

The activation of the type I and type II pathways can dictate the selection criteria for anti-cancer therapies. The mutational status of genes involved in IFN-γ signaling is a prognostic tool used to select patients that are eligible for anti-CTLA (Ipilimumab). Even if, IFN-γ alone has been tested in clinical trials in melanoma and failed (130, 131), patients harboring DNA lesions in gene encoding for IFN-γ, as well as mice carrying tumors mutated in *IFNGR1*, poorly responded to immunotherapy. Accordingly, patients treated with Ipilimumab displayed T cells with an enhanced production of IFN-γ (132).

Accordingly, in a study demonstrating the efficacy of a peptide vaccine, using the melanoma specific epitope Trp2_180_, IFN-γ reduced the capability of CD8+ T cells to recognize and kill melanoma cells. The authors demonstrated that IFN-γ increases the expression of both cognate and non-cognate MHC-I on tumor cells that can compete for the binding to TCR and limit CD8+ cells activity (133). This is a clear example on how experimental conditions, for instance the use of tumor cells instead of peptide-expressing APCs or freshly purified CD8+ T cells, can influence immune responses by shifting the balance between immune surveillance and evasion. It also explains the dual role of IFN-γ as a pro- and anti-tumor effector, depending on circumstances.

Finally, treatments of melanoma patients with anti-PD-1 (Pembrolizumab) achieved long lasting responses and recent data showed that nearly 25% of patients became refractory to immunotherapy and experienced cancer progression. The explanation of therapy resistance in these patients relies on loss-of-function mutations in JAK1 and JAK2, involved in the IFN signaling pathway (134). On the other hand, melanoma patients showing poor T cell infiltrates, do not benefit from anti-PD-1 immunotherapy. It is important to notice that when Tuting’s group used a melanoma mouse model with exiguous immune infiltrate, they observed that the stimulation of type I IFN signaling sensitizes mice to anti-PD-1 monoclonal antibody (11).

## Conclusion

Recent findings showed that type I and II IFNs are essential for tumors’ immunoediting, as is the case with CRC and melanoma. IFNs can also act directly on cancer cell behavior, having a double role in promoting proliferation or growth inhibition. Indeed, several studies established conflicting results with regard to the function of IFNs as tumor promoters or tumor suppressors in melanoma and CRC. Discrepancies can originate from different experimental settings such as the influence of the microenvironment, the quantity and quality of immune infiltrate, and the mutational status of cancer cells.

Several therapies have been elaborated to selectively target IFNs, especially IFN-α, and have obtained good clinical outcomes in melanoma patients whilst no appreciable results were obtained in the treatment of CRCs. These findings are explained by the well-high immunogenicity of melanoma and thus, its high susceptibility to be influenced by the immune system and related cytokines, such as IFNs. Indeed, melanoma harbors an elevated number of mutations, with respect to CRC, which is mirrored by the expression of aberrant proteins that serve as neo-antigens for the recognition by the immune system machinery.

Thus, there is a need to better understand the biology of IFNs in cancer and to analyze data depending on circumstances. In this context, we envision that the designing of more personalized therapies and an optimal combination of cancer vaccines, checkpoint blockade immunotherapy, cell transfer, and IFNs, will significantly contribute to the improvement of cancer patient outcomes.

## Author Contributions

SDF and AT analyzed the bibliographic data, collected them, and wrote the manuscript. MT and GS critically revised the manuscript.

## Conflict of Interest Statement

The authors declare that the research was conducted in the absence of any commercial or financial relationships that could be construed as a potential conflict of interest.
